# Role of Oil Palm Empty Fruit Bunch-Derived Cellulose in Improving the Sonocatalytic Activity of Silver-Doped Titanium Dioxide

**DOI:** 10.3390/polym13203530

**Published:** 2021-10-14

**Authors:** Yi Ding Chai, Yean Ling Pang, Steven Lim, Woon Chan Chong, Chin Wei Lai, Ahmad Zuhairi Abdullah

**Affiliations:** 1 Department of Chemical Engineering, Lee Kong Chian Faculty of Engineering and Science, Universiti Tunku Abdul Rahman, Kajang 43000, Malaysia; nicoleyd@1utar.my (Y.D.C.); stevenlim@utar.edu.my (S.L.); chongwchan@utar.edu.my (W.C.C.); 2 Centre for Photonics and Advanced Materials Research, Universiti Tunku Abdul Rahman, Kajang 43000, Malaysia; 3 Nanotechnology and Catalysis Research Centre (NANOCAT), Institute of Advanced Studies (IAS), Universiti Malaya, Kuala Lumpur 50603, Malaysia; cwlai@um.edu.my; 4 School of Chemical Engineering, Universiti Sains Malaysia, Nibong Tebal 14300, Malaysia; chzuhairi@usm.my

**Keywords:** titanium dioxide, cellulose, silver doping, sonocatalysis, Congo red

## Abstract

In this study, a novel cellulose/Ag/TiO_2_ nanocomposite was successfully synthesized via the hydrothermal method. The cellulose extracted from oil palm empty fruit bunch (OPEFB) could address the disposal issue created by OPEFB biomass. Characterization studies such as FESEM, EDX, HRTEM, XRD, FTIR, UV–Vis DRS, PL, XPS, and surface analysis were conducted. It was observed that the incorporation of cellulose could hinder the agglomeration, reduce the band gap energy to 3 eV, increase the specific surface area to 150.22 m^3^/g, and lower the recombination rate of the generated electron-hole pairs compared to Ag/TiO_2_ nanoparticles. The excellent properties enhance the sonocatalytic degradation efficiency of 10 mg/L Congo red (up to 81.3% after 10 min ultrasonic irradiation) in the presence of 0.5 g/L cellulose/Ag/TiO_2_ at 24 kHz and 280 W. The improvement of catalytic activity was due to the surface plasmon resonance effect of Ag and numerous hydroxyl groups on cellulose that capture the holes, which delay the recombination rate of the charge carriers in TiO_2_. This study demonstrated an alternative approach in the development of an efficient sonocatalyst for the sonocatalytic degradation of Congo red.

## 1. Introduction

The textile industry is the main contributor to dye pollution. The annual effluent of textile industry was estimated at approximately 100,000 tons of dyes [1]. There are more than 10,000 types of dye that are commercially available and azo dyes are the most used dye in the industry (60–70%) [2]. This is due to their simple and versatile synthesis, high dyeing ability, and chemical stability. In general, azo dyes comprise at least one azo group bonded with aromatic rings that contain functional groups such as amino, methyl, and sulfonic acids [3]. The π conjugated bonds and resonance in the aromatic structure contributed to its high stability [2]. Azo dyes are hazardous contaminants that produce mutagenic and carcinogenic compounds such as aromatic amines when released into the environment [4]. Therefore, conventional treatments are insufficient for degrading them because they are resistant to acid/alkali and light irradiation.

Advanced oxidation processes (AOPs) such as sonolysis, ozonation, and Fenton oxidation are known to generate various types of reactive oxygen species that can rapidly oxidize the contaminants. Ultrasonic irradiation has received considerable attention due to its high penetrability in liquid medium, environmental friendliness, low cost, and zero sludge formation [3,5]. Acoustic cavitation is the key factor in ultrasonic irradiation due to the generation, growth, and explosive collapse of bubbles [6]. During the collapsing of bubbles, the generated shock waves from the nanosecond implosions will increase local temperature (5000 K) and pressure (1000 atm) [5]. Water molecules are then thermally dissociated into hydroxyl (•OH) and hydrogen (H^+^) radicals. Eventually, these highly reactive radicals will attack the organic pollutants and transform them into water, carbon dioxide, and inorganic salts.

Among these semiconductors, titanium dioxide (TiO_2_) is widely used as a heterogeneous catalyst owing to its chemical properties such as low toxicity, low cost, and high chemical stability [7]. Nevertheless, TiO_2_ could not be excited by the visible light due to its wide band gap energy (3.2 eV). The rapid recombination rate of electron-hole pairs (within nanosecond) can influence its catalytic efficiency [8]. To overcome these shortcomings, TiO_2_ can be modified with metal/non-metal doping [9,10,11], semiconductor coupling [12], and semiconductor photosensitization [13]. Among these approaches, silver (Ag) doping was chosen in this study as Ag can serve as a prominent plasmonic material for sensitizing TiO_2_. Moreover, Ag is generally cheap and exhibits low toxicity and high antibacterial ability than compared to gold and platinum [14]. The incorporation of an appropriate amount of Ag can reduce the band gap energy of TiO_2_ and inhibit the recombination of electron-hole pairs by trapping the electrons due to the prominent surface plasmon resonance [15].

Recently, the conversions and utilization of agricultural biomass into higher value-added products to minimize the massive quantity of waste and to fulfill the concept of circular economy are attracting attention [16]. Agricultural biomass consists mostly of cellulose, hemicellulose and lignin. Cellulose is attractive due to its abundancy, renewability, zero toxicity, remarkable mechanical strength, biodegradability, and biocompatibility [17]. It contains several types of functional groups such as hydroxyl (–OH) and carboxyl groups. Pollutants can be adsorbed through the electrostatic interaction of the contaminant structure with these oxygen-containing functional groups. Furthermore, the addition of cellulose with high functional groups on semiconductors can increase surface area and enable well dispersion of Ag/TiO_2_ nanoparticles by reducing agglomeration. Subsequently, this introduces additional active sites and eventually enhances catalytic activity [18]. The synergistic effect of the cellulose-TiO_2_ composites has attracted the interest of researchers to develop new catalysts for the remediation of wastewater.

The present work demonstrated the investigation of oil palm empty fruit bunch (OPEFB) biomass-based cellulose/Ag/TiO_2_ composites for the enhancement of sonocatalytic degradation of Congo red for the first-time. This novel catalyst was able to address the problems faced by using the bare TiO_2_ such as wide band gap energy, rapid recombination rate of electron-hole pairs in pure TiO_2_, and agglomeration phenomenon experienced by the Ag/TiO_2_ nanoparticles. Moreover, it was reported that OPEFB is a major biomass waste in palm oil mills, reaching about 20–23% of fresh fruit bunch [19]. This had created a critical disposal issue to the environment, and the huge amount of cellulose (30–50% by weight) contained in OPEFB can serve as a potential additive material in Ag/TiO_2_ nanoparticles. The physicochemical properties of the synthesized samples were also characterized comprehensively through various types of characterization techniques such as field emission scanning electron microscopy (FESEM), energy dispersive X-Ray (EDX) analysis, high-resolution transmission electron microscopy (HRTEM), X-Ray diffraction analysis (XRD), Fourier transform infrared spectroscopy (FTIR), UV–vis diffuse reflectance spectroscopy (DRS), photoluminescence analysis (PL), X-ray photoelectron spectroscopy (XPS), and surface analysis. This was followed by catalytic performance analysis using the adsorption and sonocatalytic degradation of Congo red.

## 2. Materials and Methods

### 2.1. Materials

Sodium hydroxide (99%), n-hexane (96%), hydrochloric acid (37%), and silver sulfate (≥98.5%) were purchased from Merck (Merck KGaA, Darmstadt, Germany). Next, sodium hypochlorite (2.4%) was purchased from Clorox (Oakland, CA, USA). Titanium (IV) isopropoxide (97%) was obtained from Sigma-Aldrich (St. Louis, MO, USA). Moreover, ethanol (95%) and Congo red (40%) were bought from R&M Chemicals (Selangor, Malaysia). All received chemicals were applied without extra purification. OPEFBs were collected from a palm mill located at Selangor, Malaysia. Lastly, distilled water was utilized throughout this research study.

### 2.2. Preparation of Cellulose

OPEFB was first cut into small fragments, washed thoroughly with distilled water, and immersed in n-hexane solution for 4 h in order to remove excess palm oil. These samples were oven dried at 70 °C for 24 h and ground into OPEFB fibers (<600 µm). The OPEFB fibers were then treated with ethanol for 60 min, rinsed with distilled water, and oven-dried at 100 °C overnight to eliminate wax and other impurities. The method for cellulose extraction from OPEFB fibers was modified based on a research study conducted by Lefatshe et al. [20]. The amount of 10 g of OPEFB fibers prepared earlier was treated with 4% w/w sodium hydroxide solution at 90 °C for 2 h in an alkali treatment in order to eliminate lignin and hemicellulose. These samples were treated with 10% *w*/*w* hydrochloric acid solution at 45 °C for 2 h to undergo acid hydrolysis in order to further remove the remaining hemicellulose content. Then, the samples were bleached with 2.4 wt% sodium hypochlorite solution at room temperature for 3 h in order to enhance physical outlook and to eliminate the residual lignin and hemicellulose. Lastly, the extracted cellulose was rinsed and oven dried at 100 °C for 24 h to remove excess moisture content.

### 2.3. Preparation of TiO_2_, Cellulose/TiO_2_, Ag/TiO_2_, and Cellulose/Ag/TiO_2_

The hydrothermal synthesis conditions for TiO_2_, cellulose/TiO_2_, Ag/TiO_2_, and cellulose/Ag/TiO_2_ were modified according to the research study conducted by Zhang et al. [21]. Initially, 9 mL of titanium (IV) isopropoxide was added into 41 mL of ethanol under stirring conditions for 30 min. The resultant solution was added dropwise into a 50% *w*/*w* ethanol solution under stirring conditions for 60 min. Silver sulfate was added into 50% *w*/*w* ethanol solution according to the mass ratio of Ag: TiO_2_ equal to 0.05:1 under stirring conditions for 2 h. The Ag mixture was then added into the prepared TiO_2_ solution under stirring condition for another 30 min. Ethanol acted as a cosolvent and a reducing agent to reduce Ag^+^ to metallic Ag [22]. The amount of 0.1 M sodium hydroxide solution was added into the mixture under stirring conditions for 1 h in order to promote the reduction process [23]. The cellulose-ethanol suspension was prepared based on the mass ratio of cellulose: TiO_2_ equal to 0.05:1. Next, the Ag-doped TiO_2_ solution was added dropwise into the cellulose-ethanol solution under stirring conditions for 30 min. The final mixture was sonicated in a water bath for 30 min to minimize particle aggregation. The mixture was added into a Teflon-lined autoclave before hydrothermal synthesis. Lastly, the mixture was heated in the oven at 130 °C for 20 h. The solution was centrifuged to collect the final sample after cooling to room temperature. The final sample was oven-dried at 100 °C overnight and labelled as cellulose/Ag/TiO_2_. The same procedure was conducted to prepare cellulose/TiO_2_ at different mass ratios of 0.05:1, 0.25:1, 0.5:1, 0.9:1, and Ag/TiO_2_ at different mass ratios of 0.01:1, 0.03:1, 0.1:1, and 0.15:1. Pure TiO_2_ was also synthesized hydrothermally as control for comparison with other synthesized samples.

### 2.4. Sample Characterization

The surface morphology and elemental composition of the prepared samples (TiO_2_, cellulose, 0.05 cellulose/TiO_2_, 0.05 Ag/TiO_2_, and cellulose/Ag/TiO_2_) were studied by using FESEM (JEOL Ltd., Tokyo, Japan) and EDX (Hitachi Ltd., Tokyo, Japan), respectively. Moreover, the high-resolution images of cellulose/Ag/TiO_2_ were obtained by employing a Philips Model Tecnai 20 transmission electron microscope (Koninklijke Philips N.V., Amsterdam, Netherlands). The crystalline phase of the prepared samples was determined by using LabX XRD-6000 X-ray diffractometer (Shimadzu Corp., Tokyo, Japan) with CuKα radiation. The crystallite size was estimated by using the Debye–Scherrer equation based on the most intense peak found in the sample. A Thermo Scientific Nicolet iS10 Fourier transform infrared spectroscope (Thermo Fisher Scientific Inc., Waltham, MA, USA) was used to determine the functional groups on the catalyst. Moreover, surface analysis was conducted by utilizing a Micromeritics 3Flex surface characterization analyser (Micromeritics Instrument Corp., Norcross, GA, USA). The specific surface area of the samples was determined using the Brunauer–Emmett–Teller (BET) method based on nitrogen adsorption–desorption data at 77.35 K. Meanwhile, the pore size and pore volume were obtained based on the desorption of nitrogen isotherm using the Barrett–Joyner–Halenda (BJH) method. UV–Vis DRS was performed on the prepared samples to obtain the band gap energies by using a Perkin Elmer Lambda 35 UV–Vis spectrophotometer (PerkinElmer Inc., Waltham, MA, USA). In addition, the separation efficiencies of the generated electron-hole pairs were evaluated by using an Edinburgh FLS 920 Time Resolved Fluorescence Spectrometer (Edinburgh Instruments Ltd., Livingston, SCT, UK) with an excitation wavelength at 300 nm. An Omicron DAR 400 X-ray photoelectron spectroscope (Scienta Omicron Inc., Uppsala, Sweden) was used to investigate the surface chemical compositions and chemical and bonding states of the cellulose/Ag/TiO_2_ with monochromatized Al Kα at a binding energy of 1486.7 eV as the X-ray source.

### 2.5. Adsorption and Sonocatalytic Degradation of Congo Red

The sonocatalytic activities of the prepared samples (cellulose/TiO_2_ and Ag/TiO_2_ at different mass ratio, followed by TiO_2_, cellulose, 0.05 cellulose/TiO_2_, 0.05 Ag/TiO_2_, and cellulose/Ag/TiO_2_) were evaluated by using the removal of Congo red under ultrasound irradiation. In a typical experiment, 0.5 g/L of catalyst was added into 10 mg/L of Congo red solution to study the catalytic performance. The dye solution containing catalysts was left in the dark for 30 min of pre-adsorption to reach adsorption–desorption equilibrium. The ultrasonic irradiation with ultrasonic frequency of 24 kHz and power of 280 W was introduced to the dye solution by using a Hielscher Model UP400S ultrasonic processor. The ultrasonic probe was inserted into the dye solution to perform the sonocatalytic degradation of dye. The collected dye samples were centrifuged at 10,000 rpm for 10 min to separate the catalyst. A Jenway Model 6320D UV–Vis spectrophotometer was used to determine the residual concentration of Congo red samples at a maximum absorbance of 497 nm. The degradation efficiency of Congo red was calculated based on Equation (1):

Degradation efficiency (%) = (C_0_ ─ C_t_)/C_0_ × 100% (1)

where C_0_ is the initial dye concentration (mg/L) before sonocatalysis process, and C_t_ is the resultant dye concentration at time t. All experiments were repeated 3 times to determine the error bar for the sample data. Chemical oxygen demand (COD) test was conducted to observe the COD removal in Congo red upon 60 min of ultrasonic irradiation. No catalyst reusability and stability tests were conducted in this study as the scope was only focused on the as-prepared catalysts’ characterization and sonocatalytic performance rather than regeneration studies.

## 3. Results and Discussion

### 3.1. Characterization Study

#### 3.1.1. FESEM, HRTEM and EDX

**Figure 1:**
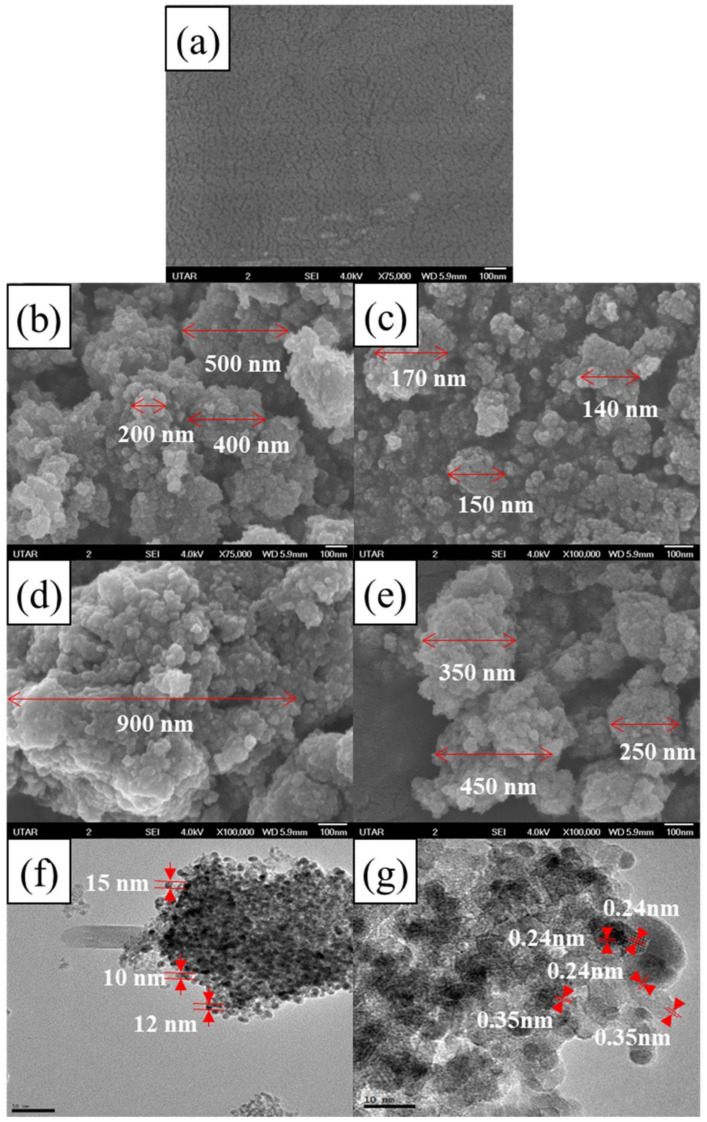
FESEM images of (**a**) cellulose, (**b**) TiO_2_, (**c**) 0.05 cellulose/TiO_2_, (**d**) 0.05 Ag/TiO_2_, and (**e**) cellulose/Ag/TiO_2_; HRTEM images of cellulose/Ag/TiO_2_ at magnifications of (**f**) 50,000× and (**g**) 100,000×.

Figure 1 shows the surface morphology of cellulose, TiO_2_, 0.05 cellulose/TiO_2_, 0.05 Ag/TiO_2_, and cellulose/Ag/TiO_2_. It was observed that the TiO_2_ nanomaterials were spherical shapes with an average size ranging from 10 to 15 nm as shown in Figure 1a. These nanoparticles were agglomerated to a size range of 200–500 nm. The formation of nanoparticles cluster was due to the reduction in surface energy to improve their stability [24]. Meanwhile, cellulose presented a smooth surface with no cracks observed, as shown in Figure 1b, which might be attributed to the hydrogen bond linkage. This result also demonstrated that the acid extraction of cellulose was unable to develop sufficient porosity on the cellulose structure. A similar finding was obtained by Zhang et al. [25], where the smooth surface of isolated cellulose from pine wood biomass was observed between the fibril bundles.

In the past, surfactants (i.e., sodium dodecyl benzene sulfonate, polyvinylpyrrolidone, 1,2,3,4-butanetetracarboxylic acid, poly(methacrylic acid), and hexadecyl trimethyl ammonium bromide) or stabilizers were added to prevent agglomeration and to control the size to achieve good dispersion of TiO_2_ nanomaterials [26]. However, rapid nucleation and slow crystal growth were observed when utilizing these surface-active materials. In this study, TiO_2_ nanoparticles were distributed on the cellulose by using a simple mixing method to intercalate the unstable TiO_2_ nanoparticles. It was anticipated that cellulose served as a carbon support material for the adherence of nanoparticles, which in turn hindered TiO_2_ agglomeration/aggregation and increased the dispersity of the nanoparticles, as shown in Figure 1c. A noticeable larger particle size of 0.05 Ag/TiO_2_ (>15 nm) was aggregated into a larger cluster of about 900 nm, as shown in Figure 1d. This revealed the strong interaction between Ag/TiO_2_ nanoparticles. Barakat et al. [27] also reported a similar result where the introduction of Ag resulted in the formation of larger aggregates of TiO_2_ particles. Meanwhile, the incorporation of cellulose into the 0.05 Ag/TiO_2_ sample demonstrated smaller aggregated nanoparticles ranging from 250 to 450 nm, as shown in Figure 1e, than compared to 0.05 Ag/TiO_2_.

Figure 1f,g show the high-resolution TEM images of cellulose/Ag/TiO_2_. The particle size of the cellulose/Ag/TiO_2_ was in the range of 10–15 nm and slightly agglomerated, as shown in Figure 1f. No clear lattice fringe can be observed for the cellulose, which suggests their amorphous carbon nature [28]. The localized doping of Ag on the surface of TiO_2_ was spotted in Figure 1g. The crystalline lattice fringe spacing of 0.35 nm corresponded to the (101) facet of the anatase phase of TiO_2_, while 0.24 nm corresponded to Ag (111) facet. These spacings could be clearly identified, as shown in Figure 1g [29]. These findings were consistent with the results obtained from the XRD analysis.

The EDX analysis was performed to quantitatively evaluate the elements present on the material. Based on the results shown in Table 1, it proved the formation of TiO_2_ after hydrothermal processes as the detected atomic ratio of Ti and O (1:2.1) was quite close to the stoichiometric composition of TiO_2_. The detected 4.54 wt% Ag element in 0.05 Ag/TiO_2_ was close to the actual amount loaded on TiO_2_ (5% wt./wt.). Singh et al. [30] reported that cellulose typically exhibited a higher percentage of carbon than compared to oxygen elements. The detected silica indicated that silica bodies were also embedded in the OPEFB fiber, while sodium and chloride elements were contributed by the alkaline treatment followed by acid hydrolysis during the extraction of cellulose.

#### 3.1.2. XRD

**Figure 2:**
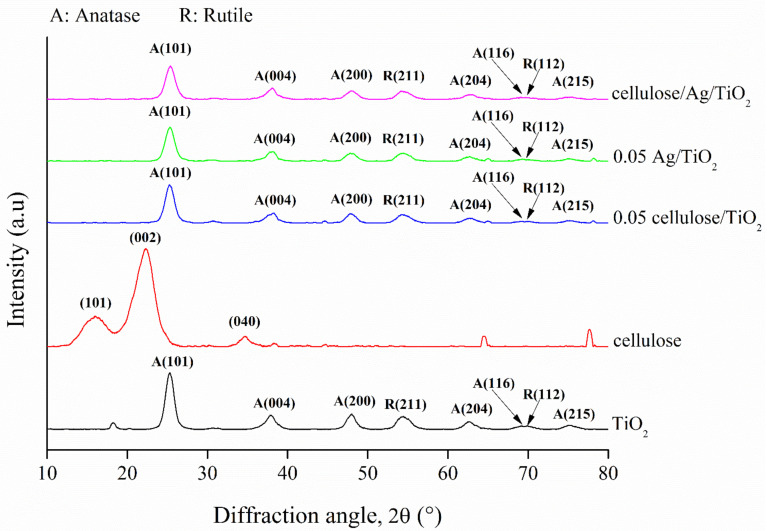
XRD patterns of TiO_2_, cellulose, 0.05 cellulose/TiO_2_, 0.05 Ag/TiO_2_, and cellulose/Ag/TiO_2_.

Figure 2 shows the XRD patterns of TiO_2_, cellulose, 0.05 cellulose/TiO_2_, 0.05 Ag/TiO_2_, and cellulose/Ag/TiO_2_. The diffraction peaks of anatase TiO_2_ displayed at 25.30, 37.88, 47.92, 62.66, 69.22, and 75.27° were attributed to the respective planes of (101), (004), (200), (204), (116), and (215) [31,32]. There were two diffraction peaks of rutile TiO_2_ found at 54.47 and 69.78°, which were indexed as (211) and (112) planes, respectively [33]. This demonstrated that the dominant phase was anatase and the minor phase was rutile in the prepared TiO_2_. Meanwhile, the diffraction peaks of cellulose appeared at 16.08, 22.26, and 34.63°, which could be contributed to the (101), (002), and (040) crystallographic planes of cellulose I polymorph [20]. Islam et al. [34] reported that the small peak at 34.5° indicated the removal of lignin and hemicellulose after chemical treatment. The results indicated the successful extraction of cellulose from OPEFB.

On the other hand, 0.05 cellulose/TiO_2_, 0.05 Ag/TiO_2_, and cellulose/Ag/TiO_2_ displayed similar diffraction peaks as the TiO_2_ sample. However, the diffraction peak intensity at 25.30° was reduced after the introduction of foreign substances such as cellulose and Ag into the prepared samples. No Ag characteristic peaks were found in 0.05 Ag/TiO_2_ and cellulose/Ag/TiO_2_, which may be ascribed to the relatively low Ag content on the surface. Komaraiah et al. [31] reported that lower amount of Ag (<5 at %) would result in a high dispersion of Ag elements on the TiO_2_ surface. They claimed that the diffraction signal for metallic Ag at 44.3° would appear only if using a higher amount of Ag doping.

Moreover, the decrement of the peaks intensity was attributed to the reduction in crystallite sizes in both 0.05 Ag/TiO_2_ and cellulose/Ag/TiO_2_. The average crystallite sizes of the synthesized samples were estimated by using Debye–Scherrer’s equation [35]. The crystallite sizes of the TiO_2_, 0.05 cellulose/TiO_2_, 0.05 Ag/TiO_2_, cellulose/Ag/TiO_2_, and cellulose were 6.55, 6.49, 5.94, 6.09, and 3.27 nm, respectively. These sizes were in good agreement with the results obtained from FESEM and HRTEM analyses as reported in the earlier section. According to Dey et al. [36], only a limited amount of Ag ions would be incorporated into the TiO_2_ lattice due to the larger ionic radius of Ag^+^ (0.126 nm) as compared to Ti^4+^ (0.068 nm). The ions would mostly accommodate themselves in the interstitial sites or deposited on the surface of TiO_2_.

#### 3.1.3. FTIR Analysis

**Figure 3:**
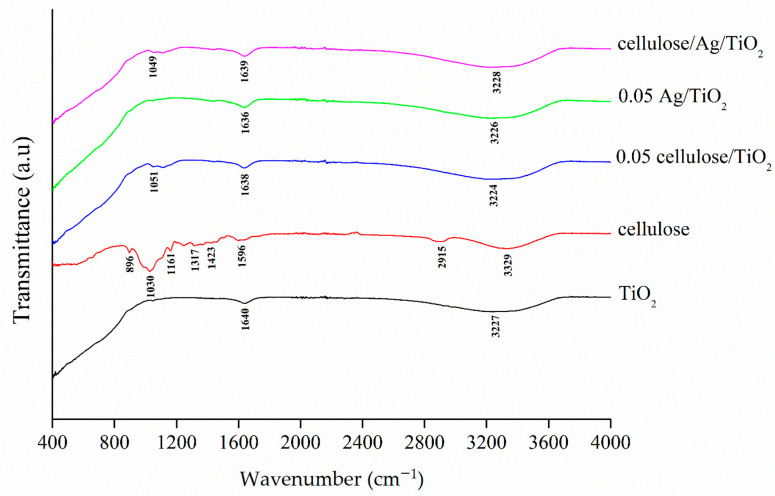
FTIR spectra of TiO_2_, cellulose, 0.05 cellulose/TiO_2_, 0.05 Ag/TiO_2_, and cellulose/Ag/TiO_2_.

Figure 3 shows the FTIR spectra for TiO_2_, cellulose, 0.05 cellulose/TiO_2_, 0.05 Ag/TiO_2_, and cellulose/Ag/TiO_2_. The broad peak between 400 cm^−1^ and 1000 cm^−1^ found in the pure TiO_2_ and TiO_2_-based samples was due to the stretching vibrations of Ti–O, Ti–O–C, and Ti–O–Ti bonds in the TiO_2_ lattice structure [37]. This implied the formation of Ti-OH bonds in all these samples.

Cellulose exhibited characteristic bands at 896 cm^−1^, 1030 cm^−1^, 1161 cm^−1^, 1317 cm^−1^, 1423 cm^−1^, and 2915 cm^−1^, which corresponded to the β-glucosidic linkages between the glucose monomers, stretching vibration of C–O–C within saccharide rings, asymmetric valence vibrations of C–O–C, bending of C–H, bending vibration of –CH_2_, and stretching vibration of C–H bonds, respectively [38,39]. All these characteristic bands corresponded to cellulose I [38,39]. The peak shifting from 1030 cm^−1^ to 1051 cm^−1^ and 1049 cm^−1^ found in 0.05 cellulose/TiO_2_ and cellulose/Ag/TiO_2_, respectively, indicated the bonding interaction between cellulose and TiO_2_ where the stretching vibration of C–O–C within saccharide rings was present.

Meanwhile, the two peaks around 3440 cm^−1^ and 1630 cm^−1^ observed in all samples were ascribed to the stretching vibration of hydroxyl groups (−OH) and adsorbed water molecules on the surface of samples, which was also reported in previous literature [40]. Both high intensity peaks at 3329 cm^−1^ and 1596 cm^−1^ found in cellulose indicated the presence of high density free hydroxyl groups and water molecules that adsorbed onto the cellulose after the removal of lignin and hemicellulose from the surface of the fibers [41]. The addition of cellulose and/or Ag into TiO_2_ increased the peaks intensity, which was related to the water molecules adsorbed by the composite materials. It should be highlighted that the presence of OH groups is beneficial for generating •OH radicals during the sonocatalysis process.

#### 3.1.4. Surface Analysis

**Figure 4:**
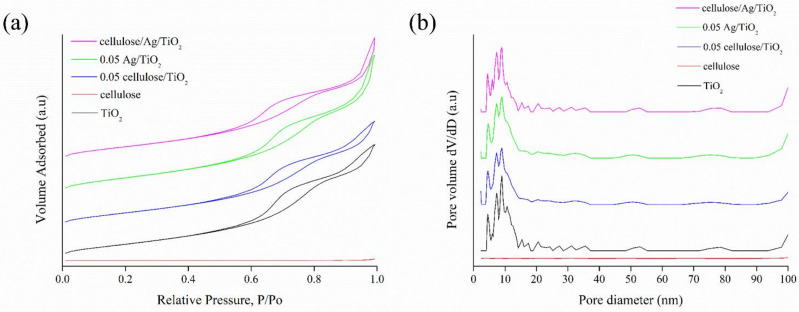
(**a**) Nitrogen adsorption–desorption plot and (**b**) pore size distribution of TiO_2_, cellulose, 0.05 cellulose/TiO_2_, 0.05 Ag/TiO_2_, and cellulose/Ag/TiO_2_.

Nitrogen adsorption–desorption isotherms were conducted to further illustrate the porosity structure of all the prepared samples. Figure 4 shows the nitrogen adsorption–desorption isotherms and pore size distributions of the samples. According to IUPAC classification [42], all samples exhibited type IV isotherms with H3 hysteresis loops, indicating the slit-like shape mesoporous characteristics of materials (2–50 nm). The dramatic increase in nitrogen adsorption at high relative pressures (above P/P_0_ = 0.6) indicated the changes of monolayer to multilayer adsorption of nitrogen followed by capillary condensation inside the mesopores [39]. The multilayer adsorption and capillary condensation happened at higher relative pressures (P/P_0_ = 0.6–0.99) further confirmed the presence of uniform mesopores, which covered the surface of TiO_2_, 0.05 cellulose/TiO_2_, 0.05 Ag/TiO_2_, and cellulose/Ag/TiO_2_. This was consistent with previous studies regarding TiO_2_ hybrid samples [43]. Alothman [44] reported that the desorption curve of H3 hysteresis consisted of a slope-related force on the hysteresis loop due to the tensile strength effect that might occur for nitrogen at 77 K and the relative pressure range of 0.4–0.45. Cellulose exhibited a horizontal trend of nitrogen adsorption and desorption at relative pressures between 0.2 and 0.9, which revealed a relatively lower external surface area [45]. Further evidence could be found from the pore size distribution shown in Figure 4b. Its external surface area was mainly constituted in the macropores regions. This feature could limit the adsorption capacity of cellulose during the adsorption followed by oxidation process. A similar low specific surface area of pristine cow dung (0.7456 m^2^/g) was also being reported by Zhu et al. [46].

Figure 4b shows the pore size distribution curves of the samples. It was observed that all samples exhibited wide mesopore sizes ranging from 3 to 50 nm. Based on Table 2, the average pore size of TiO_2_, cellulose, 0.05 cellulose/TiO_2_, 0.05 Ag/TiO_2_, and cellulose/Ag/TiO_2_ were 7.58, 26.45, 7.38, 8.83, and 8.41 nm, respectively. It was reported that mesoporous structure was beneficial for catalytic process due to its short bulk diffusion length of charge carriers and transport pathways for the diffusion of reactants [47].

As shown in Table 2, 0.05 Ag/TiO_2_ and cellulose/Ag/TiO_2_ nanocomposites had higher specific surface area and pore size than pure TiO_2_. Interestingly, the surface area of 0.05 Ag/TiO_2_ was substantially higher than that of TiO_2_, as shown in Table 2. This indicated that Ag not only served as the plasmonic light absorber but also enhanced the surface area contributed by the decrement of nanoparticles sizes of the tetragonal phase of TiO_2_ at an appropriate dopant amount [48]. The high surface area usually favors the reaction due to the presence of more active sites that can enhance the catalytic activities. It is worth noting that the surface area of cellulose/Ag/TiO_2_ decreased slightly after the incorporation of cellulose. The decrease in surface area of cellulose/Ag/TiO_2_ could be caused by blockages and coverage of cellulose on the adsorption/active sites of Ag/TiO_2_. Nevertheless, Ng and Leo [39] mentioned that cellulose could promote the dispersion of Ag/TiO_2_ nanoparticles and helped to reduce particle agglomeration without affecting the specific surface area.

It was reported that large BET surface area was beneficial for the dispersion of active species (i.e., low valance Ti species and oxygen vacancies) and allowed higher exposure to the organic dye adsorption followed by oxidation [49]. The larger mesopores would also accelerate the diffusion of reactants and products, which contributed to higher catalytic performance. The incorporation of Ag nanoparticles also contributed to the growth of pore size and pore volume in 0.05 Ag/TiO_2_ and cellulose/Ag/TiO_2_. The formation of larger mesopores pore size, pore volume, and specific surface area of cellulose/Ag/TiO_2_ was expected to promote the performance of cellulose/Ag/TiO_2_ during the degradation of dye pollutants.

There was also a slight decrease in the specific surface area, pore size, and pore volume of 0.05 cellulose/TiO_2_ with respect to the bare TiO_2_ counterpart. Oliveira et al. [50] reported a similar decrement in surface area for cellulose-TiO_2_ hybrids at various cellulose loadings. They claimed that it might be related to the partial blockage of the mesopore structure in TiO_2_ by the cellulose entities.

#### 3.1.5. Optical Properties

**Figure 5:**
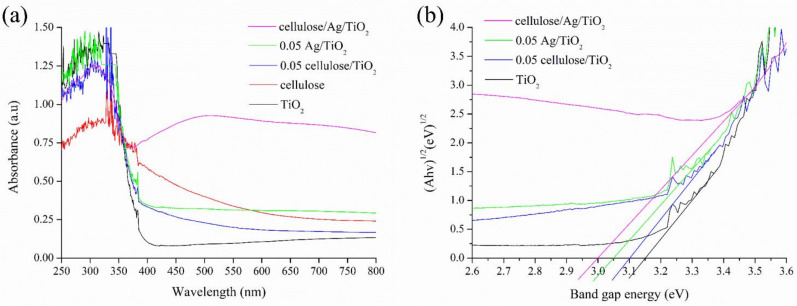
(**a**) Optical properties and (**b**) band gap energy of the prepared samples.

The optical properties of TiO_2_, cellulose, 0.05 cellulose/TiO_2_, 0.05 Ag/TiO_2_, and cellulose/Ag/TiO_2_ were determined using UV–Vis DRS, and the results are shown in Figure 5a. TiO_2_ showed absorption in the ultraviolet (UV) region (200 to 400 nm) and absorption edge at approximately 400 nm due to the intrinsic band gap transition from the valence band to the conduction band of TiO_2_ and the existence of rutile phase [51,52]. Meanwhile, 0.05 cellulose/TiO_2_, 0.05 Ag/TiO_2_, and cellulose/Ag/TiO_2_ showed higher absorption properties due to the Schottky heterojunction formed between Ag or cellulose and TiO_2_ [52]. The optical absorption edge for TiO_2_-based materials was shifted from the UV range into visible range (i.e., red shift) of cellulose/Ag/TiO_2_, which implied the synergistically effect of the Ag or cellulose material that enhanced the absorption of visible light. Yang and Luo [51] reported that the extension of visible light absorption to above 500 nm might be due to the local surface plasmon resonance effect of the metallic species. They also claimed that Ag nanoparticles could induce TiO_2_ valence band edges to absorb visible light.

This red shift of light absorption suggested a decrement in the band gap energy as shown in Figure 5a. The band gap energies of the prepared samples were determined by employing the Tauc’s plot, as shown in Equation (2) [40]:

(2) $$\alpha h\nu =A{\left(h\nu -E_{g}\right)}^{n/2}$$

where *hν* is the incident photon energy, *α* is the absorption coefficient, *A* is a constant, and *E_g_* is the band gap energy (eV). The value of *n* = 4 represents the indirect transition. Figure 5b presents the plots of (Ahν)^1/2^ versus band gap energy. The E_g_ values of TiO_2_, 0.05 cellulose/TiO_2_, 0.05 Ag/TiO_2_, and cellulose/Ag/TiO_2_ were about 3.15, 3.1, 3.05, and 3 eV, respectively. Xue et al. [53] claimed that the lower band gap energies of nanocomposite samples than compared to bare TiO_2_ were due to the surface plasmon effect of Ag^0^. Zhou et al. [40] reported that the formation of Schottky junction at the interface of Ag nanoparticles and TiO_2_ could capture the generated electrons and resulted in the accumulation of redundant electrons on Ag nanoparticles. This inhibited the recombination rate of electron-hole pairs and, subsequently, enhanced light response and catalytic activity. The lower band gap energies findings confirmed the enhancement of light utilization efficiency for the nanocomposite materials.

#### 3.1.6. PL

**Figure 6:**
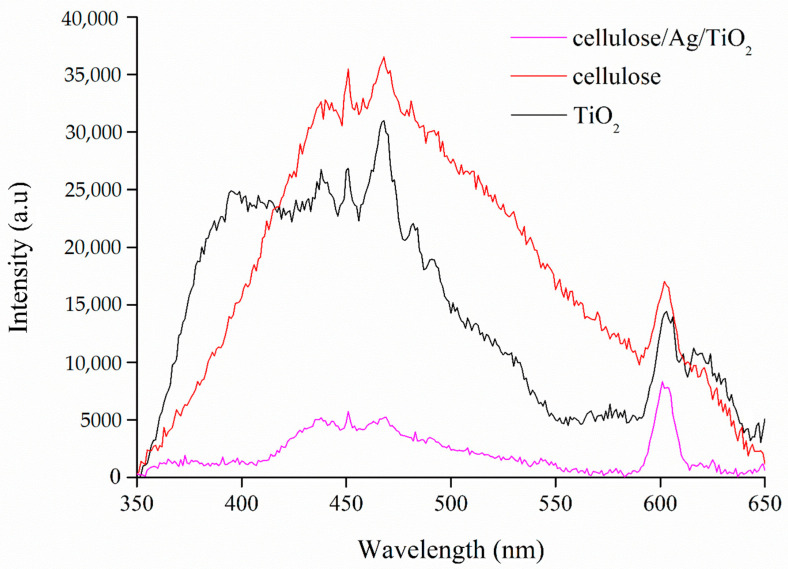
PL spectra of TiO_2_, cellulose, and cellulose/Ag/TiO_2_.

Photoluminescence (PL) measurements were performed to study the radiative recombination of photo-induced electrons and holes that emit fluorescence, and the results are shown in Figure 6. High PL intensity usually denotes a strong recombination rate of charge carriers and vice versa. The irregular shapes of the PL spectrum for TiO_2_ indicated that the sample exhibited several emission peaks. The PL bands might be assigned to the conductor and valence bands transition (399 nm) [54], the free excitons of band edge (450 nm) [55], surface oxygen vacancies associated with Ti^3+^ in anatase (438, 490, 540, and 606 nm) [31,55], the crystal lattice defects (480 nm) [55], recombination of the photo-induced electron–hole pair (468 nm) [55], and the transition between the gap state and valence band (620 nm) [54].

As shown in Figure 6, the fluorescence emission spectrum of cellulose exhibited emission peaks at about 440, 481, and 602 nm when excited at 300 nm. Liu et al. [56] reported that the unique emission behaviors of cellulose may originate from the electron-rich oxygen and/or glucose units, which also confirms the aggregation-induced or crystallization-induced emissions from cellulose. Shanthini et al. [57] also revealed that cellulose consisted of two intense PL bands at about 420–440 nm and 480–490 nm. On the other hand, the decreasing PL intensity in cellulose/Ag/TiO_2_ was attributed to a decrease in the recombination rate of charge carriers in TiO_2_ and the improvement of charge separation. Mahnae et al. [58] reported that Ag nanoparticles could act as surface trap centers for emitting electrons, and this could decrease the PL intensity of cellulose/Ag/TiO_2_. Consequently, generated electrons and holes in TiO_2_ had a high probability to react with oxygen and water molecules, respectively, to produce highly reactive oxygen species that were capable in degrading organic pollutants via oxidation processes, as highlighted by Sboui et al. [59].

#### 3.1.7. XPS

**Figure 7:**
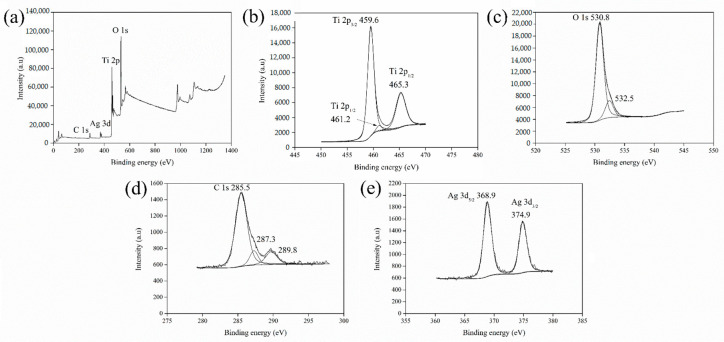
XPS spectra of cellulose/Ag/TiO_2_: (**a**) survey, (**b**) Ti 2p peaks, (**c**) O 1s peaks, (**d**) C 1s peaks, and (**e**) Ag 3d peaks.

XPS analysis was employed to detect the elemental composition and chemical states in cellulose/Ag/TiO_2_. The wide scanning XPS spectrum shown in Figure 7a demonstrated that the surface elemental composition for cellulose/Ag/TiO_2_ comprised 25.71% Ti, 61.02% O, 12.22% C, and 1.05% Ag. Figure 7b shows the high-resolution XPS spectra of the Ti 2p and the two characteristic peaks located at binding energies of 459.6 and 465.3 eV. They were corresponded to Ti 2p_3/2_ and Ti 2p_1/2_, which were in agreement with the reported literature data for the anatase phase [60,61]. The obtained energy difference of 5.7 eV in the Ti 2p doublet due to spin-orbit splitting also confirmed the existence of Ti^4+^ species in TiO_2_ [62].

A weak Ti 2p_1/2_ peak located at 461.2 eV might be attributed to the Ti^3+^ sites in TiO_2_ lattice, which indirectly demonstrated the presence of oxygen vacancies [63]. The abundant hydroxyl groups in cellulose played an important role in the reduction of Ti^4+^ to Ti^3+^ during the hydrothermal process [64]. Fu et al. [65] reported that the C-OH in the alcohol (i.e., ethanol and isopropanol) from the hydrolysis of titanium isopropoxide could reduce Ti^4+^ to Ti^3+^. On the other hand, Wang et al. [66] reported that the minority of Ti^3+^ species (Ti^3+^ 2p_1/2_ and 2p_3/2_) might be related to a redox reaction involved in TiO_2_ and monovalent Ag(I) species, Ag^+^ + Ti^3+^ → Ag^0^ + Ti^4+^. It was reported that the Ti^3+^ and oxygen vacancies could build a new energy level and hinder the recombination of electrons and holes [67]. The oxygen vacancy sites were more likely to adsorb oxygen molecules to form chemisorbed oxygen species, such as superoxide radicals (O^2–^).

In the O1s spectrum shown in Figure 7c, the two peaks formed at binding energies of 530.8 and 532.5 eV were associated with the lattice oxygen in TiO_2_ (O–Ti–O bond) and chemisorbed water molecules or carbonyl groups from cellulose (Ti–O–H or Ti–O–C), respectively [41,59]. The surface adsorbed oxygen would react with the generated electrons to produce anion radicals. Meanwhile, the reactive and unstable –OH groups would react with the generated holes to produce •OH radicals [68].

Figure 7d shows the high-resolution XPS spectrum of C 1s in cellulose/Ag/TiO_2_, which exhibits three splitting peaks at 285.5, 287.3, and 289.8 eV. The carbon peak located at 285.5 eV was attributed to the C–C or C–H bonds originating from a surface layer of adventitious carbon [69,70]. The carbon peak occurred at 287.3 eV was associated with the O–C–O or C–OH bonds [71,72]. Another carbon peak formed at 289.8 eV was attributed to carbonyl, aldehydic, or ketonic carbon on the surface of cellulose/Ag/TiO_2_ [41]. This peak at 289.8 eV was also associated with the carboxylic or carbonate groups generally present on the surface of the metal oxides [51,73].

The high-resolution XPS spectrum of Ag 3D region peaked at 368.9 and 374.9 eV, as shown in Figure 7e. The binding energies of 368.9 and 374.9 eV corresponded to Ag 3d_5/2_ and Ag 3d_3/2_, respectively [74]. The obtained 6 eV of energy spacing between these two peaks confirmed the characteristics of Ag nanoparticle whereby Ag existed in the form of Ag^0^ [75]. The presence of Ag^0^ indicated that the Ag ions were reduced to Ag atoms in cellulose/Ag/TiO_2_.

### 3.2. Catalytic Performance

**Figure 8:**
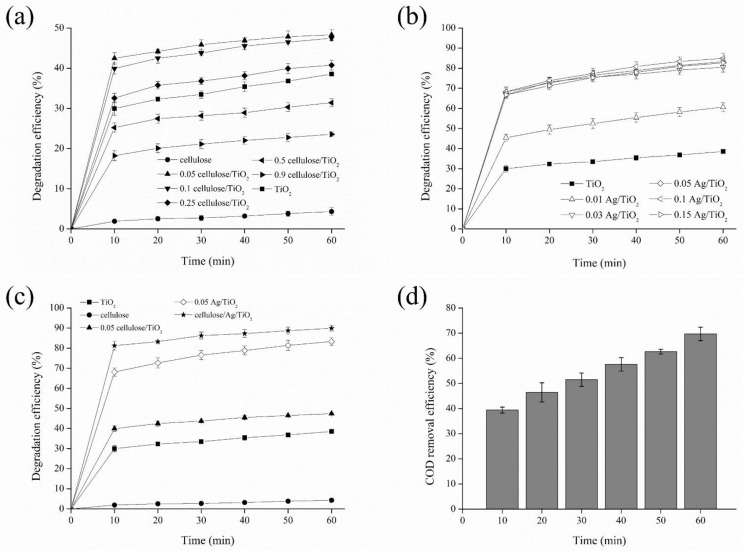
Degradation efficiency of Congo red for (**a**) cellulose/TiO_2_ composites, (**b**) Ag/TiO_2_ composites, (**c**) cellulose/Ag/TiO_2_, and their (**d**) COD removal efficiencies under ultrasonic irradiation (initial dye concentration = 10 mg/L, catalyst loading = 0.5 g/L, treatment time = 60 min, ultrasonic frequency = 24 kHz, and power = 280 W).

The sonocatalytic activities of different mass ratios of cellulose/TiO_2_ were studied, and the results are shown in Figure 8a. It was found that the sonocatalytic degradation efficiency of Congo red was extremely slow and negligible (4.3% after 60 min) in the presence of cellulose alone. The sonocatalytic degradation efficiency of Congo red increased to 48.3% when utilizing an appropriate amount of cellulose incorporated into TiO_2_ (0.05 cellulose/TiO_2_). A decreasing trend in the sonocatalytic performance of Congo red was observed when increasing the amount of cellulose beyond the optimum mass ratio value. This might be related to the increasing contact area and surface exposition of cellulose entities in cellulose/TiO_2_ [50]. The surface adsorption sites for dye molecules on cellulose allowed the neighboring TiO_2_ to interact with the adsorbed dye molecules easily. This subsequently facilitated their interactions with the generated electrons-holes carriers for oxidation process to occur. Although 0.05 cellulose/TiO_2_ exhibited slightly lower surface area value (142.06 m^2^/g) than compared to TiO_2_ (146.46 m^2^/g), its interfaced network and abundant –OH groups endowed within 0.05 cellulose/TiO_2_ served as a better catalyst compared to TiO_2_ itself.

Voisin et al. [76] reported that the excellent adsorption properties of nanocellulose for various pollutants could contribute to the high catalytic activity of the hybrid materials by trapping the dye molecules close to the active centers. Based on the XRD analysis, the average crystallite size of 0.05 cellulose/TiO_2_ (6.49 nm) was smaller than pure TiO_2_ (6.55 nm). Nsib et al. [77] reported that the small crystallite size could give rise to a large number of small particles, which acted as reactive sites for the reaction of the holes with the H_2_O and –OH groups adsorbed at the TiO_2_ surface to generate •OH radicals. Moreover, the formation of organic–inorganic inter-penetrating networks could also create more –OH groups on the cellulose surface and contribute to the formation of more •OH radicals than compared to bare TiO_2_. These •OH radicals produced by the 0.05 cellulose/TiO_2_ composite were important for reacting with the adsorbed dye molecules and formed lower molecular weight degradation products.

Figure 8b shows the effect of different mass ratio of Ag/TiO_2_ on the sonocatalytic degradation efficiency of Congo red. The sonocatalytic degradation efficiency of Congo red initially increased from 60.7% to 80.5% when increasing the mass ratio of Ag: TiO_2_ from 0.01:1 to 0.03:1. The sonocatalytic performance only increased insignificantly when further increasing the mass ratio of Ag: TiO_2_ to 0.1:1. An appropriate amount of Ag nanoparticles could enhance the catalytic performance of TiO_2_ through charge transferring, electron trapping, and reduction in band gap energy [78]. Yang and Luo [51] reported that an excessive amount of Ag metal might form a charge trapping center for electron-hole pairs, restrain the movement of charge carriers, and consequently reduce catalytic efficiency. Although 0.1 Ag/TiO_2_ achieved the highest sonocatalytic performance among the Ag-doped TiO_2_ samples, 0.05 Ag/TiO_2_ (83.3%) with the catalytic performance close to 0.1 Ag/TiO_2_ (84.9%) was selected for the subsequent study after considering the lower impact on catalyst development cost.

Figure 8c shows the sonocatalytic degradation efficiency of Congo red in the presence of various types of catalysts. The results showed the insignificant sonocatalyic degradation of Congo red in the presence of cellulose. Lan et al. [79] reported that cellulose has no capability of absorbing the visible light and showed insignificant absorption of UV light (<250 nm). Meanwhile, the sonocatalytic degradation efficiency of Congo red in the presence of cellulose/Ag/TiO_2_ significantly increased to 89.9%. Ahmadi et al. [3] reported that the small particle size of the catalyst could increase the surface area available for degradation of organic pollutants. The smaller particle size and larger specific surface area found in cellulose/Ag/TiO_2_ than compared to other types of catalysts provided additional active sites, which promoted the mass transfer of dye pollutants between the liquid and the catalyst surface. The synergistic effect between Ag, cellulose, and TiO_2_ nanoparticles might be related to the generation of numerous •OH radicals that promoted electron transfer ability, the reduction in recombination rate of charge carriers, and the decrease in band gap energy in cellulose/Ag/TiO_2_. Subsequently, the sonocatalytic performance of Congo red improved by 51.4% in the presence of cellulose/Ag/TiO_2_ than compared to bare TiO_2_. Therefore, cellulose/Ag/TiO_2_ was determined as a suitable catalyst to effectively degrade Congo red under ultrasonic irradiation.

The high degradation efficiency of Congo red was insufficient to indicate the high mineralization of Congo red dye molecules into the final products of carbon dioxide and water. COD analysis is necessary to study the mineralization of Congo red. Figure 8d shows the COD removal efficiency of Congo red in the presence of cellulose/Ag/TiO_2_. It was found that the COD removal efficiency of Congo red was 39.4% at the first 10 min and eventually increased to 69.7% after ultrasonication for 60 min. The increasing trend in COD removal efficiency was due to the formation of reactive free radicals that promoted the redox reactions for Congo red degradation during ultrasonic irradiation. Compared to degradation efficiency, a lower COD removal efficiency of Congo red indicated that the intermediate organic byproducts remained in the treated solution and mineralization was incomplete. The low mineralization of Congo red might also be related to the degraded aromatic related molecules that inhibited Congo red from being adsorbed onto the surface of cellulose/Ag/TiO_2_ during ultrasonic irradiation [80].

In order to evaluate the performance of sonocatalytic degradation of Congo red using cellulose/Ag/TiO_2_, the obtained results were compared with those reported in the literature and shown in Table 3. The obtained results demonstrated that sonocatalytic degradation of 10 mg/L of Congo red in the presence of cellulose/Ag/TiO_2_ achieved 81.2% in a shorter time period of 10 min. This concluded that the performance of cellulose/Ag/TiO_2_ at relatively lower catalyst amounts used in this study significantly surpassed those cited in the literature in terms of sonocatalytic degradation efficiency.

### 3.3. Sonocatalytic Degradation Mechanisms of Congo Red

**Figure 9:**
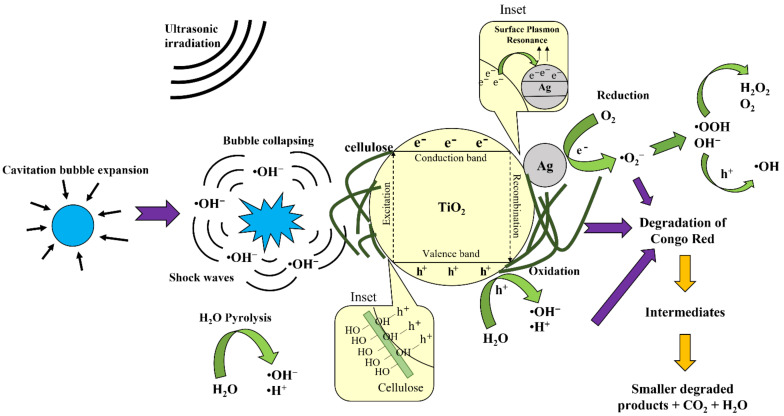
Proposed sonocatalytic degradation mechanism of Congo red with cellulose/Ag/TiO_2_.

Figure 9 shows the proposed degradation mechanism of Congo red in the presence of cellulose/Ag/TiO_2_ upon ultrasonic irradiation. Firstly, ultrasonic irradiation is introduced to the Congo red solution in the presence of cellulose/Ag/TiO_2_. Ultrasonic irradiation triggered the formation of cavitation bubbles [85]. During ultrasonic irradiation, cellulose/Ag/TiO_2_ nanoparticles imparted more nucleation sites and increased the occurrence of cavitation bubbles [86]. These bubbles subsequently underwent formation, oscillation, growth, and finally collapsed. When the cavitation bubbles collapsed, the large amount of energy released would generate instantaneous hot spots with high temperature and pressure. These hot spots facilitated the thermal dissociation of water and oxygen by producing •OH and H^+^ radicals (Equation (3)) [3]. These free radicals consisted of unpaired electrons that were highly reactive and possessed strong oxidation abilities [85].

H_2_O + ))) (ultrasound) → •OH + H^+^ (3)

Meanwhile, sonoluminescence occurred during the collapse of cavitation bubbles and emitted an instantaneous flash of light [87]. This flash light was able to excite electrons from the conduction band and left holes at the valence band of TiO_2_ (Equation (4)) [86]. The generated electrons reduced the oxygen molecules to form superoxide (•O_2_^−^) radicals (Equation (5)), while the generated holes (h^+^) oxidized the water molecules to form •OH radicals (Equation (6)). The generated •O_2_^−^ radicals further reacted with the water molecules to form hydroperoxyl (•OOH) radicals and hydroxide (OH^−^) ions, which resulted in the production of hydrogen peroxide (H_2_O_2_) molecules (Equations (7)–(8)) [51]. The reaction of OH^−^ ions with the generated holes formed •OH radicals (Equation (9)).

cellulose/Ag/TiO_2_ + hν → cellulose/Ag/TiO_2_ (h^+^ + e^−^) (4)

O_2_ + e^−^ → •O_2_^−^ (5)

H_2_O + h^+^ → •OH + H^+^ (6)

•O_2_^−^ + H_2_O → •OOH + OH^−^ (7)

2•OOH → H_2_O_2_ + O_2_ (8)

OH^−^ + h^+^ → •OH (9)

The Schottky barrier was created on the Ag/TiO_2_ interface due to the lower Fermi level of Ag than compared to the conduction band of TiO_2_, where the conduction band edge positions for TiO_2_ and Ag were estimated to be −0.11 V and +0.16 V vs. SHE, respectively [88]. Such close contact between TiO_2_ and Ag in the Ag/TiO_2_ formed the Fermi level alignment to realize a p-n-type heterojunction. Upon excitation, the generated electrons would transfer from conduction band TiO_2_ to the Ag particles. The conduction band of Ag nanoparticles served as a trap center for the generated electrons and inhibited the electron-hole recombination rate. Another possible reason was related to the localized surface plasmon resonance effect in Ag nanoparticles, which enabled sonoluminescence light absorption by localized surface plasmon excitation [20]. This would generate electrons and occupy energy levels above the Fermi level of the Ag, thereby restraining the recombination of generated electron-hole pairs onto the surface of TiO_2_ [15]. Thus, both electrons from surface plasmon resonance excited Ag nanoparticles and the electrons transferred from TiO_2_ to Ag might be captured by the adsorbed O_2_ on the catalyst surface to form radical superoxide anions. Subsequently, more reactive radicals were formed to accelerate and oxidize Congo red to intermediate organic byproducts and, finally, into carbon dioxide and water.

Meanwhile, cellulose played an important role in cellulose/Ag/TiO_2_ composite during sonocatalysis reaction even though it did not possess any catalytic degradation ability. Innumerable –OH groups existing on the cellulose surface would improve the adsorption capacity in Congo red pollutants removal and captured the holes upon light excitation of TiO_2_. Eventually, the electron and hole pairs could be separated, and the reaction between holes and –OH groups would generate •OH radicals, which was beneficial for the subsequent oxidation reactions in the mineralization process of Congo red. Moreover, Yang and Luo [51] also found that the carbon sites in cellulose behaved similarly to an electron acceptor center for trapping the electrons emitted from the conduction band of TiO_2_ and eventually enhanced charge separation efficiency. These generated electrons would migrate to the surface of the particles to undergo reduction processes and produced reactive species such as superoxide ions, which stimulated the radical chain reactions. In short, cellulose was able to assist in the separation of electrons and holes in cellulose/Ag/TiO_2_ composites and achieved a high participation rate of charge carriers in the sonocatalytic reaction.

## 4. Conclusions

In the present work, cellulose/Ag/TiO_2_ with the mass ratio of cellulose: Ag: TiO_2_ = 0.05:0.05:1 was successfully prepared through the incorporation of OPEFB-derived cellulose on Ag-doped TiO_2_ via hydrothermal synthesis. The physicochemical properties of TiO_2_, 0.05 cellulose/TiO_2_, 0.05 Ag/TiO_2_, and cellulose/Ag/TiO_2_ were studied through FESEM, EDX, HRTEM, XRD, FTIR, UV–Vis DRS, PL, XPS, and surface analysis. The spherical morphology was not affected in TiO_2_-based samples upon preparation. The OPEFB-derived cellulose exhibited cellulose I structure, whereas bare TiO_2_ and TiO_2_-based samples displayed major anatase and minor rutile phases. The band gap energy and recombination rate of charge carriers in TiO_2_ was significantly reduced due to Ag doping. Improvements of the surface properties of cellulose/Ag/TiO_2_ such as large mesopores pore size, pore volume, and specific surface area promoted the sonocatalytic performance of cellulose/Ag/TiO_2_. The sonocatalytic degradation efficiency of Congo red successfully achieved 89.9% in the presence of cellulose/Ag/TiO_2_ at optimum conditions (catalyst loading = 0.5 g/L, initial dye concentration = 10 mg/L, treatment time = 60 min, ultrasonic frequency = 24 kHz, and power = 280 W). The synergetic effect of cellulose improved the catalytic activity of Ag/TiO_2_, which prolonged the lifetime of the hole and electron pairs to participate in redox reactions. This study has proven the usefulness of biomass-derived cellulose in the development of an efficient sonocatal yst.

## Figures and Tables

**Table 1:** EDX analysis of TiO_2_, cellulose, 0.05 cellulose/TiO_2_, 0.05 Ag/TiO_2_, and cellulose/Ag/TiO_2_.

Element/Samples	TiO_2_		Cellulose		0.05 Cellulose/TiO_2_		0.05 Ag/TiO_2_		Cellulose/Ag/TiO_2_	
	Weight %	Atomic %	Weight %	Atomic %	Weight %	Atomic %	Weight %	Atomic %	Weight %	Atomic %
Ti	58.64	32.13	-	-	49.55	23.71	56.41	32.17	42.59	19.60
O	32.13	67.87	46.10	40.03	41.99	60.15	39.05	66.68	40.29	55.52
C	-	-	50.45	58.35	8.46	16.15	-	-	19.60	24.06
Ag	-	-	-	-	-	-	4.54	1.15	4.00	0.82
Si	-	-	0.54	0.27	-	-	-	-	-	-
Na	-	-	0.99	0.60	-	-	-	-	-	-
Cl	-	-	1.93	0.76	-	-	-	-	-	-

**Table 2:** Surface analysis of TiO_2_, cellulose, 0.05 cellulose/TiO_2_, 0.05 Ag/TiO_2_, and cellulose/Ag/TiO_2_.

Sample	Pore Size (nm)	Pore Volume (cm^3^/g)	Specific Surface Area (m^2^/g)
TiO_2_	7.58	0.3287	146.46
cellulose	26.45	0.0038	0.88
0.05 cellulose/TiO_2_	7.38	0.3043	142.06
0.05 Ag/TiO_2_	8.83	0.3828	157.58
cellulose/Ag/TiO_2_	8.41	0.3251	150.22

**Table 3:** Comparison of cellulose/Ag/TiO_2_ performance with other methods for sonocatalytic degradation of Congo Red.

Type of Catalyst	Concentration (mg/L)	Catalyst Loading (g/L)	Ultrasound Power (W)	Treatment Time (min)	Degradation (%)	Ref
KNbO_3_	5	1.0	300	300	69.23	[81]
TiO_2_	10	1.0	50	120	25.69	[82]
TiO_2_	10	1.5	50	180	100	[83]
SnO_2_/CdSe/Bi_2_O_3_	10	1.0	300	150	100	[84]
cellulose/Ag/TiO_2_	10	0.5	280	10	81.2	Present work
				60	89.9	

## Data Availability

The data presented in this study are available on request from the corresponding author.
